# Lockdown measures for COVID-19 outbreak and variation in physical activity in patients with heart failure and cardiac implantable devices

**DOI:** 10.1016/j.ijcha.2021.100906

**Published:** 2021-10-28

**Authors:** Pedro Silva Cunha, Sérgio Laranjo, André Lourenço, Lourenço Rodrigues, Isabel Cardoso, Guilherme Portugal, Bruno Valente, Ana Sofia Delgado, Rui Cruz Ferreira, Ana Abreu, Mário Martins Oliveira

**Affiliations:** a Arrhythmology, Pacing and Electrophysiology Unit, Cardiology Service, Santa Marta Hospital, Central Lisbon Hospital University Centre, Portugal; b Cardiovascular Rehabilitation Center, Faculty of Medicine, University of Lisbon, Portugal; c ISEL, Instituto Superior de Engenharia de Lisboa, Portugal

**Keywords:** Remote monitoring, Carelink™ network, Heart failure, Physical activity

## Abstract

**Aims:**

The present study analysed the patterns of physical activity pre-, during and post-lockdown measures for COVID-19 pandemic in patients with chronic heart failure (CHF) and cardiac implantable electronic devices (CIED) under remote monitoring (RM), and assessed the physical activity patterns during these periods.

**Methods:**

The raw data from 95 patients with CHF (age 67,7 ± 15,1 years, 71,5% male) corresponding to 2238 RM transmissions of the Medtronic Carelink™ network platform was obtained. The physical exercise profiles and the impact of the lockdown measures on the physical behaviour during and after the measures were analysed. According to the level of activity duration in the pre-lockdown, lockdown and post-lockdown periods, the patterns of behaviour were identified (non-recoverees, incomplete recoverees, recoverees and full-recoverees).

**Conclusion:**

RM of CHF patients with CIED using the Carelink™ network is useful for close follow-up and identification of heart failure risk status variations. After relieving the confinement measures there were two groups of patients that did not recover the previous physical activity levels. Physical inactivity in patients with CHF can have a significant impact on outcomes.

## 1 Introduction

A pneumonia of unknown cause detected in Wuhan, China, was first reported to the World Health Organization Country Office in China on 31 December 2019. The outbreak was declared a Public Health Emergency of International Concern on 30 January 2020. The measures imposed to various degrees across the continents have drastically restricted freedom of movement and outlawed public gatherings. On March 19, in Portugal, an emergency of public health was declared, and restrictive lockdown measures were implemented by the government to control the spread of the disease.

There have been several indirect consequences of this pandemic, like the reduction in physical activity of patients with chronic heart failure (CHF). Sedentary behaviour and physical inactivity have been considered leading modifiable risk factors for cardiovascular disease and all-cause mortality and are important lifestyle risk factors with an impact in the prognosis of CHF [1]. Remote monitoring (RM) of patients with CIED is presently routine in many centres. Current‐generation cardiovascular implantable electronic devices (CIED) can continuously monitor patient activity through built‐in accelerometers, resulting in an overview of the degree of physical activity variations during a particular time period [2] and this technology has been previously validated [3], [4], [5], [6], [7]. Medtronic ICD or CRT devices contain a single-axis accelerometer that records the number of daily minutes in which the patient exceeded an activity level equivalent to 70–80 steps per Minute [8]. The data are stored in the device for a rolling 14- month period and can be extracted.

The degree and patterns of disruption on the physical activity of patients with CHF and left ventricular dysfunction related to the lockdown period during the COVID-19 pandemic and specifically the data regarding physical activity reestablishment after suspension of the restriction measures are currently unknown. Although it is unclear how to manage comprehensively patients based on this information, the evidence obtained could be important for decision-making and prognostic evaluation.

## 2 Aims

The purpose of our study was to evaluate the direct impact of the lockdown measures on the physical activity levels and vital signs of an ambulatory cohort of CHF outpatients with an implantable cardioverter-defibrillator (ICD) or cardiac resynchronization device (CRT) followed in a RM programme. Additionally, we aimed to assess the post-lockdown behaviour of this cohort of patients and to explore whether RM can play a role in the management of this population during outstanding lockdown conditions, discouraging outpatient-clinic visits.

## 3 Methods

### 3.1 Study population

We conducted a retrospective review of the medical records of consecutive patients who were followed-up at the arrythmology RM clinic (480 patients under active follow-up) and selected patients with CIED from the ambulatory Medtronic CareLink™ RM programme (Medtronic, Minneapolis, Minnesota, USA) with at least 12 months of follow-up and had the raw-data available (95 patients). Daily physical activity data from the Medtronic ICD/CRT devices were extracted through Medtronic proprietary conversion of Carelink® interrogation data into an analysable format, and the company made these unprocessed data available to our unit. All the device transmissions were examined and the raw data obtained was analyzed through a custom-made Python script. The study was conducted in accordance with the Declaration of Helsinki and each participant provided informed consent prior to participation.

Patients were considered eligible for inclusion in our analysis if they met the following criteria: non-homebound, age over 18 years, and if they were implanted with a CIED (either ICD or CRT system, single chamber/dual chamber or biventricular) compatible with the CareLink^TM^ Patient Management system from Medtronic. All subjects had a primary diagnosis of CHF, and had been under optimal medical management previous to device implantation. Data on baseline information, hospital admissions, and cardiovascular and all cause events were collected through chart reviews.

### 3.2 Study design

Demographic data, clinical outcomes and the raw data extracted from the Medtronic Carelink™ network were entered into a database consisting of all patients.

To investigate the effects of lockdown and to detect potential variations across the study period, individual patient’s data were split into three different time series of six weeks of duration. The first time series corresponding to the lockdown period (confinement, from the 19th of March 2020 until the 2nd of May 2020), the second time series corresponding to the six weeks before lockdown (baseline, from 7th of February 2020 until the 18th of March 2020), and the third time series corresponding to the six weeks after lockdown (post-confinement, from the 3rd of May 2020 until the 14th of June 2020). During the lockdown, a general duty of home retreat was imposed, with people not being allowed to circulate in public spaces and roads, except for a few selected exceptions.

Our main purpose was to analyse the physical activity profiles of this group of patients, and the impact that the lockdown measures had on the activity level behaviour during and after the restrictive measures. Additionally, data on arrhythmic events, thoracic impedance (Optivol®), night heart rate, HRV, percentage of biventricular pacing, atrial tachycardia/atrial fibrillation (AT/AF) burden, ventricular rate during AT/AF, and appropriate and inappropriate ICD therapies were prospectively entered into a database consisting of all patients.

For this analysis, individual patient data were aggregated and summarized to derive three central tendency measures for each variable, one for each period of analysis. The classification of patient physical activity level was initially based on the baseline activity levels of all population under analysis. Quartiles were defined for the minutes of daily activity and each patient was then assigned to a quartile per the average number of minutes of daily activity in the baseline period.

### 3.3 Endpoints

The primary endpoint of the study was the variation of physical activity levels between baseline, confinement and post-confinement periods. Physical activity levels were derived from Medtronic Carelink™ Cardiac Compass Activities of Daily Living parameter, a trend of the 7-days average of accelerometer-based data.

Secondary outcomes included the change of the different clinical variables, namely: thoracic impedance (Optivol®) and average heart rate.

## 4 Statistical analysis

We investigated the statistical evidence of physical activity differences among the three analysis periods (baseline, confinement and post-confinement) and the evaluation of the patterns of evolution of the clinical variables (Optivol™, night heart rate and HRV) along the time.

Statistical analyses were performed using the Python, version 3.9.2. All analyses were done with the use of open-source packages, including Pandas version 1.2.2 (https://pandas.pydata.org), NumPy version 1.20.0 (https://numpy.org), SciPy version 1.6.1 (https://scipy.org) and ploty version 4.14.3 (https://plotly.com/python/). Before analysis, quality control was performed, and outliers were excluded. The statistical differences between each pair of periods (baseline, confinement and post-confinement) were tested. The normality of each population was tested with a Kolmogorov-Smirnov test (with α = 0.05). A repeated measures analysis of variance (ANOVA) was then used to evaluate the existence of relevant changes in activity between the different periods of time, for each quartile of physical activity.

Comparisons between quartiles of physical activity (0–3) over time (base vs. confinement vs. post-confinement) for physical activity levels were examined via two-way repeated measures analysis of variance (ANOVA). Post-hoc comparisons were performed via Bonferroni’s tests. To model the behaviour of each group of patients/ quartile of physical activity, in the different periods of time, linear mixed models were created. These models defined minutes of activity as the dependent variable and the week number as the independent one. Variability between individuals in each group was accounted by defining the subjects as random effect groups. Different models were produced for each of the time periods to understand if there existed clear and abrupt changes in physical activity behaviour motivated by the implementation and removal of confinement measures. Categorical variables were expressed as numbers and percentages and were compared with the use of Fisher’s exact or ordinal chi-square tests, as appropriate. Continuous variables were expressed as means and standard deviations and were compared with the use of paired Student’s *t*-test. All p-values were based on two-sided tests.

## 5 Results

### 5.1 Baseline characteristics

A total of 95 CHF patients with CIED were included (10% in NYHA class I, 53% in class II, 36% in class III and 1% in ambulatory class IV), mean age 67,7 years, 71,5% male. Ischemic CHF was present in 47% of the patients and AF in 24,1%. Mean baseline left ventricular ejection fraction (LVEF) was 32,7%. An ICD was implanted in 59 patients (62%), a CRT defibrillator (CRT-D) was implanted in 30 patients (32%) and a CRT pacemaker (CRT-P) in the remaining (6%). The characteristics of the overall population at baseline are summarized in Table 1. Mean follow-up time was 47 months ±19months, with no patients lost to follow-up. The analysis was focused on 2238 transmissions via the RM system.

**Table 1:** Patients characteristics.

	Total
**Total number of patients, n**	95
**Age, years (mean ± SD)**	67,1 ± 14,1
**Male, n (%)**	68 (71,5)
**Aetiology**	
Ischaemic, n (%)	45 (47)
Dilated cardiomyopathy, n (%)	37 (39)
Valvular, n (%)	3 (3)
Congenital, n (%)	5 (5)
Hypertrophic, n (%)	5 (5)
**Ejection Fraction, % (mean ± SD)**	32,7 ± 11,9
**HF characteristics**	
NYHA class at implantation, n (%)	
I	10 (10)
II	50 (53)
III	34 (36)
IV	1 (1)
**Type of CEID**	
ICD	59 (62)
CRT-D	30 (32)
CRT-P	6 (6)
**Hypertension, n (%)**	52 (54,7)
**Diabetes, n (%)**	26 (27,3)
**Atrial Fibrillation, n (%)**	23 (24,2)
**CKD, n (%)**	18 (18,9)
**OSAS, n (%)**	7 (7,3)
**COPD, n (%)**	5 (5,2)

Legend. HF, heart failure; NYHA, New York Heart Association; COPD, chronic obstructive pulmonary disease; CKD, chronic kidney disease; OSAS, obstructive sleep apnea syndrome. CIED, cardiac implantable electronic devices; ICD, Implantable cardioverter‐defibrillator; CRT-D, Cardiac resynchronization therapy with ICD; CRT-P, Cardiac resynchronization therapy with pacing

### 5.2 Activity level patterns

**Fig. 1:**
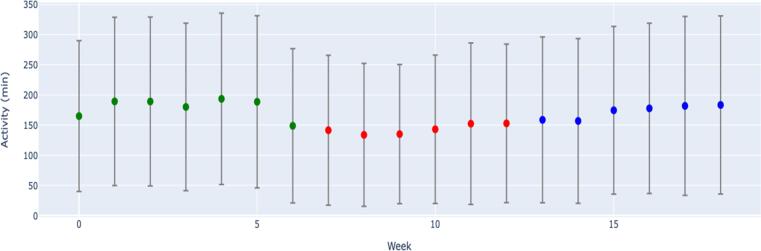
Global physical activity during the study period. Activity is represented as duration in minutes (mean ± SD) of active physical effort/ day represented along the weeks. In green the baseline period, red the confinement period and in blue the post-confinement period.

A statistically significant decrease in physical activity levels due to lockdown could be documented for all patients when compared to the baseline period (active time per day 181,93 min vs. 142,73 min; p < 0.0001) (Fig. 1).

Subsequently, four different patterns of physical activity could be established. These patterns emerged from identifying individuals by their baseline level of activity, associating each individual to the quartile of minutes of daily activity. The baseline physical activity quartiles were determined after analysing all the population, estimating the interquartile thresholds as were defined below:

Quartile 0 – less than 76 min of activity/day
Quartile 1 – a range of activity from 76 to 160 min /day
Quartile 2 – a range of activity from 161 to 254 min /day
Quartile 3 – more than 254 min of activity/day

The statistical evaluation of the distributions produced by such grouping, in the different periods of analysis, allowed further understanding of activity patterns present in this population, identifying four different groups of patients.

**Fig. 2:**
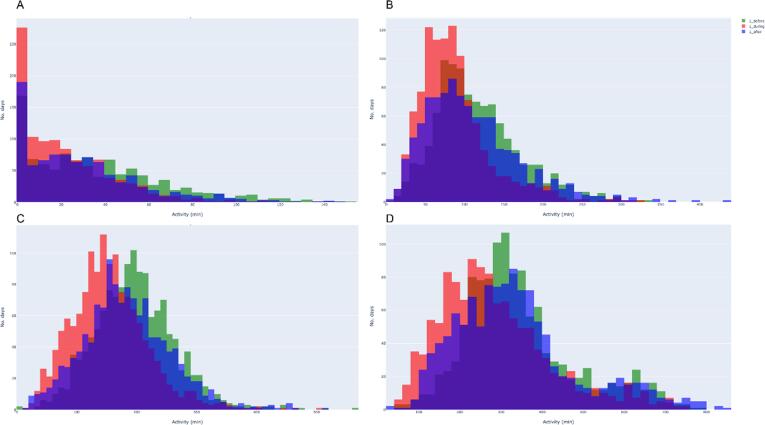
Histograms of activity grouped by baseline activity level. Legend: A- Quartile 0, B – Quartile 1, C- Quartile 2, D – Quartile 3. green - activity before lockdown; red - physical activity during the lockdown; B: blue - the level of activity after the lockdown. X axis: activity (minutes), Y axis: number of days.

**Fig. 3:**
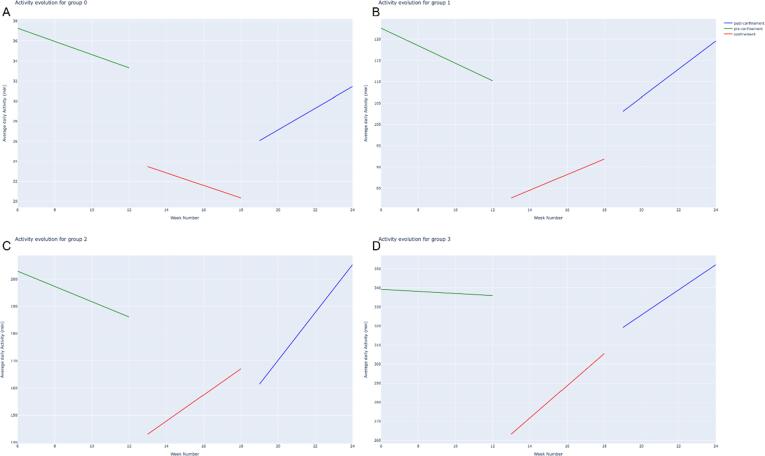
Linear mixed model for physical activity, depicting the evolution of the four groups (quartile 0 to 3), by activity level in the three periods analysed. A: After confinement, this group of patients did not recover their activity to the levels pre-confinement (“non-recoverees”). B. this group recovered, but to levels less than pre-confinement (“incomplete-recoverees”). C: this group recovered the activity to levels equivalent to pre-confinement (“recoverees”). D: After confinement, this group of patients augmented the physical activity and even surpassed the pre-confinement degree of activity (“full-recoverees”).

In the first group of patients (quartile 0, N = 24), physical activity was already severely reduced before lockdown (less than 76 min of activity/day), with a further reduction during the lockdown period that persisted throughout the post-confinement period (“non-recoverees”, Fig. 2). The second group (quartile 1, N = 23) includes patients with a sharp decrease in physical activity during lockdown, but in whom an incomplete recovery (not reaching baseline levels) was seen throughout the post-confinement period (“incomplete recoverees”, Fig. 3a). In group 3 (quartile 2, N = 25) we found patients in whom the lockdown period induced only a slight to moderate reduction in physical activity, and then recovered to activity levels slightly reduced, when compared with the baseline period (“recoverees”, Fig. 3b). Patients of group 4 (quartile 3, N = 23), with the highest levels of baseline physical activity (>254 min of activity/day), had a significant reduction of activity during confinement, but showed a full recovery post-confinement, in some cases to levels above baseline (“full recoverees”, Fig. 3c).

Accordingly, a marked decrease in physical activity from the baseline to the confinement period was documented for all groups, with significant differences between baseline and post confinement period documented only for the first two groups (Table 2).

**Table 2:** Physical activity during the study period grouped by quartiles of baseline activity.

Activity	Average	SD	Delta to baseline
**Quartile0**			
Baseline	36,59	31,31	
Confinement	21,70	21,04	−69%*
Post-Confinement	23,13	26,55	−58,19%*

**Quartile1**			
Baseline	116,81	53,42	
Confinement	87,28	46,14	−33,83%*
Post-Confinement	108,76	60,33	−7,40%

**Quartile2**			
Baseline	197,09	67,39	
Confinement	164,42	63,60	−19,87%
Post-Confinement	195,10	72,72	−1,02%

**Quartile3**			
Baseline	345,68	134,51	
Confinement	300,71	136,57	−14,95%
Post-Confinement	350,66	149,93	1,42%

*P < 0,01

The same finding could be seen in the linear mixed models analysis (Fig. 3). In all groups, it is possible to observe a small decline of activity in the six weeks preceding confinement, potentially justified by the increasing awareness of the evolution of the pandemic. Nonetheless, the slope of the models during that period does not explain the activity levels for the same groups during confinement, where, in all cases, there is a significate reduction in activity, as seen by the gaps between before and during confinement models. Finally, there is evidence for a group dependent recovery capability. Lower quartiles (Fig. 3A), corresponding to patients with low levels of activity during baseline period, continue decreasing their activity during confinement, and have slower recoveries, failing to regain their former activity. The other groups manage to recover even during confinement, with increasing results, where quartile 1 (Fig. 3B) only slightly recovers, but manages to almost achieve the same levels of activity as before confinement during the period posterior to lockdown, and quartiles 2 (Fig. 3C) and 3 (Fig. 3D) fully recovering, and exceeding the original level respectively. The main difference between these two latter groups was the period in which activity increase occurred the most: while in Quartile 2 it was after the lockdown that activity was fully recovered, Quartile 3 had practically fully recovered by the end of the confinement period.

The patient characteristics for each quartile of baseline clinical activity are summarized in Table 3. Briefly, patients from Quartile 0 were older, had a significantly lower baseline ejection fraction and higher levels of brain natriuretic peptide (BNP) when compared to the patients of Quartile 2 and 3.

**Table 3:** Patients’ characteristics grouped by quartile of baseline physical activity.

	Quartile 0	Quartile 1	Quartile 2	Quartile 3
**Patients, *n***	24	23	25	23
**Age, years (mean ± SD)**	75,6 ± 4,6	68,37 ± 11,4	60,2 ±13,7	62,4 ±12,5
**Male gender, n(%)**	15(62,5%)	18(78,2%)	17 (68%)	18 (78,2%)
**HF etiology**				
Dilated cardiomyopathy, *n* (%)	9 (37,5%)	10 (43,4%)	8 (32%)	10 (43,4%)
Ischaemic, *n* (%)	12 (50,0%)	9 (39%)	14 (56%)	10 (43,5%)
Valvular, *n* (%)	1 (4,2%)		2 (8%)	
**LVEF at implantation, %** **(mean ± SD)**	27,9 ± 5,4	31,7 ± 11,5	32,2 ± 10,4	36,4 ± 12,9
**NYHA class at implantation, *n* (%)**				
I		1 (4,2%)	3 (12%)	6 (26%)
II	8 (33,3%)	13 (56,5%)	17 (68%)	12 (52,2%)
III	15 (62,5%)	9 (39%)	5 (20%)	5 (21,7%)
IV	1 (4,2%)			
**BNP at implantation, pg/ml ()**	482,0 ± 543,3	325 ± 314,2	289,3 ± 221,4	128,7 ± 100,1
**Hypertension, n (%)**	15 (62,5%)	13 (56,5%)	14 (56%)	10 (43,5%)
**Diabetes, n (%)**	8 (33,3%)	6 (26%)	8 (32%)	4 (17,3%)
**Atrial Fibrillation, n (%)**	6 (25%)	6 (26%)	5 (20%)	6 (26%)
**COPD, n (%)**	3 (12,5%)	1 (4,3%)	1 (4%)	
**CKD, n (%)**	8 (33,3%)	5 (21,7%)	4 (16%)	1 (4,3%)
**OSAS, n (%)**	1 (4,2%)	1 (4,3%)	3 (12%)	2 (8,7%)

Legend: HF, heart failure; LVEF, left ventricular ejection fraction; NYHA, New York Heart Association; BNP, Brain Natriuretic Peptide; COPD, chronic obstructive pulmonary disease; CKD, chronic kidney disease; OSAS, obstructive sleep apnea syndrome.

### 5.3 Clinical variables

**Fig. 4:**
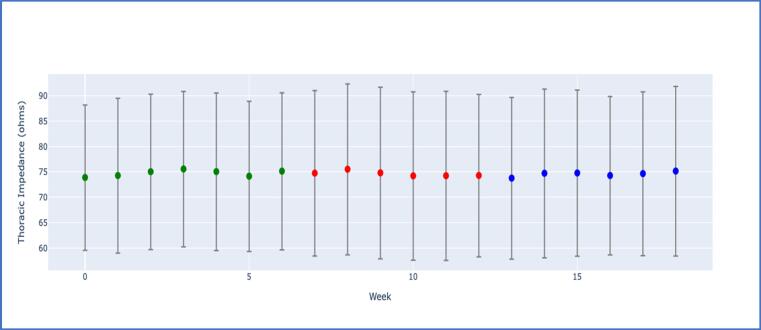
Thoracic impedance (OptiVol™) levels along the weeks. In green the baseline period, red the confinement period and in blue the post-confinement period.

**Fig. 5:**
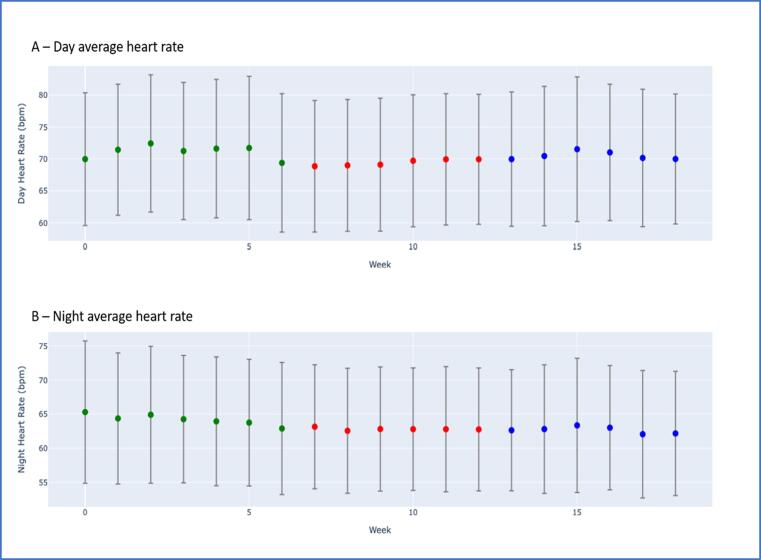
Day and night average heart rate along the weeks. A non-significant trend for progressive reduction of the heart rate could be found. In green the baseline period, red the confinement period and in blue the post-confinement period.

**Fig. 6:**
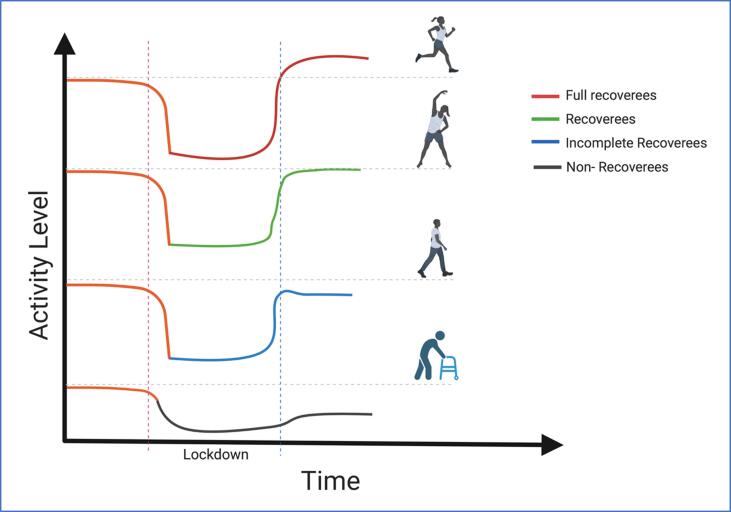
Visual abstract of the physical activity behaviour (pre-lockdown, during lockdown and after lockdown).

Interestingly, no statistically significant difference could be found between baseline, confinement and post-confinement periods for any of the other clinical variables analysed, namely: arrhythmic events; thoracic impedance (Fig. 4), HRV and AT/AF burden (Table 4). A non-significant trend for a reduction in day and night average heart rate could be documented (Fig. 5). In Fig. 6 it is represented the physical activity behaviour per-quartile (pre-lockdown, during lockdown and after lockdown).

**Table 4:** Summary of Clinical Variables.

**Daily Physical Activity (minutes)**	Average	SD	Median	IQR
Baseline	181,93	139,35	160,00	179,00
Confinement	142,73	124,55	115,00	149,00
Post-Confinement	172,10	142,19	146,00	180,00

**Day Heart Rate**				
Baseline	71,31	10,80	71,00	15,00
Confinement	69,39	10,31	69,00	14,00
Post-Confinement	70,51	10,74	70,00	15,00

**Night Heart Rate**				
Baseline	64,06	9,59	63,00	12,00
Confinement	62,80	9,10	62,00	12,00
Post-Confinement	62,66	9,29	62,00	12,00

**Daily Impedance (ohms)**				
Baseline	74,81	15,22	73,00	18,06
Confinement	74,63	16,50	72,25	19,50
Post-Confinement	74,54	16,20	72,50	19,94

**Time in AT/AF (sec)**				
Baseline	2452669,06	13220682,46		
Confinement	2946218,18	15550214,51		
Post-Confinement	2491144,30	14145910,26		

**Legend:** AT, Atrial Tachycardia; AF, Atrial Fibrillation.

## 6 Discussion

Currently, new remote management strategies are in use to follow-up patients with CHF with the objective of promptly identify and act to decrease hospitalizations [9], [10]. This type of technology is reliable, enabling early identification of device malfunction, arrhythmic events and HF decompensation, while reducing the risk of under-reporting and the number of outpatient clinic visits [11], [12]. RM should account for components of the pathophysiology contributing to decompensation [13]. Temporal changes in physiological parameters may precede signs and symptoms of worsening HF [14] allowing the health team to anticipate preventive measures, and to generate predictive models that can identify patients who are at high or low risk of clinical events [15]. Several studies have proved the utility of RM in the reduction of mortality in patients with CIED’s [16], [17], [18]. This can lead to a comprehensive approach in which knowledge of the patient's physiological status can anticipate therapeutic measures in order to avoid or minimize decompensation of HF.

One indirect consequence of the pandemic on routine medical care is that regular examination of patients and even the supply of drugs is altered due to the disturbance in the routine clinical practice. In addition, containment measures have reduced the normal mobility of patients, which may have a significant impact on clinical evolution.

Curiously, in our cohort, the previously more active patients managed not to completely reduce their physical activity during confinement. This fact could be the object of a more detailed assessment in the future. Although, we can speculate that, in part, it is because this quartiles correspond to a population with a younger age (the mean age in Quartile 2 is 60,2 ±13,7 and Q 3 is 62,4 ±12,5, compared with a mean age of 75,6 ± 4,6 in Quartile 0 and a mean age of 68,37 ± 11,4 Quartile 1). This fact is certainly of importance and may correspond to patients still maintaining some of their professional and domestic tasks, and eventually maintenance of some degree of exercise at home.

Even brief periods of exposure to these behaviors can be deleterious [19]. A study, that analyzed a 2-week reduction in daily steps from 10,501 ± 808 to 1,344 ± 33 steps/day, found that this reduction led to impaired insulin sensitivity and lipid metabolism, increased visceral fat and decreased fat-free mass and cardiovascular fitness in healthy adults [20].

Several small studies [21], [22], have analyzed the physical activity variation in patients with CIED, during the lockdown. This analysis confirmed that there is a substantial reduction in physical activity. One study found that there was a 27.1% decline in physical activity, and the median physical activity of patients significantly declined from 2.4 to 1.8 h/day [23].

Our analysis is the first to analyze comparatively the patterns of recovery after the restraint measures have been lifted.

In this sense, it is important to design home-based physical exercise programs and keep this population regularly monitored, particularly concerning the level of physical activity. Exercise is an important therapeutic intervention in CHF. Also, it has been demonstrated that in patients with CHF, physical inactivity is associated with nearly twice all‐cause and cardiac mortality, and even modest exercise is associated with a survival benefit [24], [25].

There is evidence supporting that feedback provided via the telephone and mail by medical staff may be as effective as the more traditional follow-up program in reducing symptoms and improving quality of life [26], and in decreasing rehospitalization rates [27].

In this study we tried to highlight the fact that the reduction of physical exercise during the COVID-19 in patients with CHF is significant, may be continuously monitored, and represents an alert for possible worsening of the patient's clinical status, leading to believe that intervention must be carried out to correct it. Even in a situation of profound social mobility limitation physical activity should be encouraged and specific home-based physical exercise programs can be developed for patients with CHF.

Prevention of a decompensation in CHF is an important issue, and, therefore, we should encourage our patients to maintain their activity as long as possible. If risk factors like sedentary behaviour and physical inactivity are not successfully corrected, both short- and long-term outcomes in CHF patients will likely be affected. Maximization of wellbeing and health status of CHF population is essential to prevent decompensation and promote quality of life.

Study limitations

Rigorous data collection and evaluation in the current study were employed for internal evaluation of our RM service at this institution. This is a retrospective study, with a small number of patients and without randomization, with patients being their own control. Because of the importance of identifying a significant reduction in physical activity without full recovery in a large number of cases, we did not prolong the study in order to evaluate the full impact on future clinical events. Monitoring of the magnitude of event-rates will certainly contribute to understand the dynamics of lockdown measures, inactivity and outcomes in this group of severe disease patients.

## 7 Conclusion

Implementing a RM program of CHF patients in a real-world setting of a comprehensive HF-management program is efficient. The originality of this work is the identification of the patterns of physical activity (“recoverees”, “full-recoverees”, “non-recoverees”, “incomplete recoverees”) after a period of profound inactivity in patients with CHF. This finding could be applicable to the various occasions of forced reduction of activity (like in an admission to the hospital) and be used as a parameter in future studies.

## Grant support

This work did not received any grants

## Ethical disclosures

Protection of human and animal subjects.

The authors declare that no experiments were performed on humans or animals for this study.

## Confidentiality of data

The authors declare that they have followed the protocols of their work center on the publication of patient data.

## Right to privacy and informed consent

The authors declare that no patient data appear in this article.

## Declaration of Competing Interest

The authors declare that they have no known competing financial interests or personal relationships that could have appeared to influence the work reported in this paper.
