# Supplementary material for: Lockdown measures for COVID-19 outbreak and variation in physical activity in patients with heart failure and cardiac implantable devices
Source: Int J Cardiol Heart Vasc. 2021 Oct 28;37:100906. doi: 10.1016/j.ijcha.2021.100906 (PMC8552563; doi:10.1016/j.ijcha.2021.100906)
Supplement: Supplementary data 1 [file mmc1.docx]

(0.0, 'blue') - quartile 0; post confinement

Mixed Linear Model Regression Results

===================================================================

Model: MixedLM Dependent Variable: ActivitiesOfDlyLiving

No. Observations: 131 Method: REML

No. Groups: 23 Scale: 57.1789

Min. group size: 1 Log-Likelihood: -489.3929

Max. group size: 6 Converged: Yes

Mean group size: 5.7

---------------------------------------------------------------------

Coef. Std.Err. z P>|z| [0.025 0.975]

---------------------------------------------------------------------

Intercept 5.590 9.336 0.599 0.549 -12.708 23.887

weeknumber 1.077 0.391 2.753 0.006 0.310 1.844

Group Var 386.018 17.315

===================================================================

(0.0, 'green') - quartile 0; pre confinement

Mixed Linear Model Regression Results

===================================================================

Model: MixedLM Dependent Variable: ActivitiesOfDlyLiving

No. Observations: 168 Method: REML

No. Groups: 24 Scale: 198.4338

Min. group size: 7 Log-Likelihood: -712.5938

Max. group size: 7 Converged: Yes

Mean group size: 7.0

---------------------------------------------------------------------

Coef. Std.Err. z P>|z| [0.025 0.975]

---------------------------------------------------------------------

Intercept 41.240 6.574 6.273 0.000 28.355 54.124

weeknumber -0.661 0.543 -1.217 0.224 -1.726 0.404

Group Var 434.792 10.445

===================================================================

(0.0, 'red') - quartile 0; confinement

Mixed Linear Model Regression Results

===================================================================

Model: MixedLM Dependent Variable: ActivitiesOfDlyLiving

No. Observations: 168 Method: REML

No. Groups: 24 Scale: 101.2338

Min. group size: 7 Log-Likelihood: -659.9452

Max. group size: 7 Converged: Yes

Mean group size: 7.0

---------------------------------------------------------------------

Coef. Std.Err. z P>|z| [0.025 0.975]

---------------------------------------------------------------------

Intercept 31.629 6.849 4.618 0.000 18.204 45.053

weeknumber -0.628 0.388 -1.618 0.106 -1.389 0.133

Group Var 297.955 9.865

===================================================================

(1.0, 'blue') - quartile 1; post confinement

Mixed Linear Model Regression Results

===================================================================

Model: MixedLM Dependent Variable: ActivitiesOfDlyLiving

No. Observations: 146 Method: REML

No. Groups: 25 Scale: 523.4435

Min. group size: 2 Log-Likelihood: -698.9058

Max. group size: 6 Converged: Yes

Mean group size: 5.8

---------------------------------------------------------------------

Coef. Std.Err. z P>|z| [0.025 0.975]

---------------------------------------------------------------------

Intercept 40.346 25.679 1.571 0.116 -9.984 90.676

weeknumber 3.301 1.115 2.961 0.003 1.116 5.486

Group Var 2128.040 30.950

===================================================================

(1.0, 'green') - quartile 1; pre confinement

Mixed Linear Model Regression Results

===================================================================

Model: MixedLM Dependent Variable: ActivitiesOfDlyLiving

No. Observations: 174 Method: REML

No. Groups: 25 Scale: 1024.5892

Min. group size: 6 Log-Likelihood: -866.1466

Max. group size: 7 Converged: Yes

Mean group size: 7.0

---------------------------------------------------------------------

Coef. Std.Err. z P>|z| [0.025 0.975]

---------------------------------------------------------------------

Intercept 134.959 12.297 10.975 0.000 110.857 159.060

weeknumber -2.063 1.219 -1.693 0.091 -4.452 0.326

Group Var 637.997 7.637

===================================================================

(1.0, 'red') - quartile 1; confinement

Mixed Linear Model Regression Results

===================================================================

Model: MixedLM Dependent Variable: ActivitiesOfDlyLiving

No. Observations: 173 Method: REML

No. Groups: 25 Scale: 348.9797

Min. group size: 5 Log-Likelihood: -785.6858

Max. group size: 7 Converged: Yes

Mean group size: 6.9

---------------------------------------------------------------------

Coef. Std.Err. z P>|z| [0.025 0.975]

---------------------------------------------------------------------

Intercept 58.829 12.585 4.675 0.000 34.163 83.495

weeknumber 1.835 0.715 2.566 0.010 0.433 3.237

Group Var 1016.770 17.777

===================================================================

(2.0, 'blue') - quartile 2; post confinement

Mixed Linear Model Regression Results

===================================================================

Model: MixedLM Dependent Variable: ActivitiesOfDlyLiving

No. Observations: 185 Method: REML

No. Groups: 31 Scale: 926.8852

Min. group size: 5 Log-Likelihood: -926.8224

Max. group size: 6 Converged: Yes

Mean group size: 6.0

---------------------------------------------------------------------

Coef. Std.Err. z P>|z| [0.025 0.975]

---------------------------------------------------------------------

Intercept -5.234 29.255 -0.179 0.858 -62.572 52.104

weeknumber 8.770 1.316 6.664 0.000 6.191 11.350

Group Var 1591.241 16.194

===================================================================

(2.0, 'green') - quartile 2; pre confinement

Mixed Linear Model Regression Results

===================================================================

Model: MixedLM Dependent Variable: ActivitiesOfDlyLiving

No. Observations: 227 Method: REML

No. Groups: 33 Scale: 2001.8980

Min. group size: 4 Log-Likelihood: -1196.1057

Max. group size: 7 Converged: Yes

Mean group size: 6.9

---------------------------------------------------------------------

Coef. Std.Err. z P>|z| [0.025 0.975]

---------------------------------------------------------------------

Intercept 219.704 14.369 15.291 0.000 191.543 247.866

weeknumber -2.804 1.495 -1.875 0.061 -5.734 0.127

Group Var 478.088 4.642

===================================================================

(2.0, 'red') - quartile 2; confinement

Mixed Linear Model Regression Results

===================================================================

Model: MixedLM Dependent Variable: ActivitiesOfDlyLiving

No. Observations: 227 Method: REML

No. Groups: 33 Scale: 695.4610

Min. group size: 3 Log-Likelihood: -1107.5658

Max. group size: 7 Converged: Yes

Mean group size: 6.9

---------------------------------------------------------------------

Coef. Std.Err. z P>|z| [0.025 0.975]

---------------------------------------------------------------------

Intercept 80.509 15.073 5.341 0.000 50.967 110.051

weeknumber 4.808 0.879 5.467 0.000 3.084 6.531

Group Var 1695.811 18.369

===================================================================

(3.0, 'blue') - quartile 3; post confinement

Mixed Linear Model Regression Results

===================================================================

Model: MixedLM Dependent Variable: ActivitiesOfDlyLiving

No. Observations: 168 Method: REML

No. Groups: 28 Scale: 3000.3765

Min. group size: 6 Log-Likelihood: -951.4884

Max. group size: 6 Converged: Yes

Mean group size: 6.0

---------------------------------------------------------------------

Coef. Std.Err. z P>|z| [0.025 0.975]

---------------------------------------------------------------------

Intercept 194.119 57.962 3.349 0.001 80.517 307.722

weeknumber 6.583 2.475 2.660 0.008 1.733 11.433

Group Var 14314.501 80.442

===================================================================

(3.0, 'green') - quartile 3; pre confinement

Mixed Linear Model Regression Results

===================================================================

Model: MixedLM Dependent Variable: ActivitiesOfDlyLiving

No. Observations: 198 Method: REML

No. Groups: 29 Scale: 4062.9305

Min. group size: 2 Log-Likelihood: -1135.5715

Max. group size: 7 Converged: Yes

Mean group size: 6.8

---------------------------------------------------------------------

Coef. Std.Err. z P>|z| [0.025 0.975]

---------------------------------------------------------------------

Intercept 342.519 26.607 12.873 0.000 290.370 394.668

weeknumber -0.547 2.272 -0.241 0.810 -5.001 3.907

Group Var 7977.951 38.856

===================================================================

(3.0, 'red') - quartile 3; confinement

Mixed Linear Model Regression Results

===================================================================

Model: MixedLM Dependent Variable: ActivitiesOfDlyLiving

No. Observations: 196 Method: REML

No. Groups: 28 Scale: 2385.9474

Min. group size: 7 Log-Likelihood: -1084.0941

Max. group size: 7 Converged: Yes

Mean group size: 7.0

---------------------------------------------------------------------

Coef. Std.Err. z P>|z| [0.025 0.975]

---------------------------------------------------------------------

Intercept 153.200 33.544 4.567 0.000 87.456 218.945

weeknumber 8.464 1.745 4.852 0.000 5.044 11.883

Group Var 11991.864 74.063

===================================================================
